# Entscheidungsansatz zwischen Plattenosteosynthese versus Endoprothese bei proximalen Humerusfrakturen des rheumatischen Schultergelenks

**DOI:** 10.1007/s00393-025-01656-6

**Published:** 2025-05-14

**Authors:** Christoph Biehl, Anna-Catarina Meyer, Maren Seidl, Christian Heiß, Markus Rupp, Thaqif El Khassawna

**Affiliations:** 1 Klinik und Poliklinik für Unfall‑, Hand- und Wiederherstellungschirurgie – Operative Notaufnahme, Labor für Experimentelle Unfallchirurgie, Universitätsklinikum Gießen und Marburg GmbH, Rudolf-Buchheim-Str. 7, 35385 Gießen, Deutschland; 2 Experimental Trauma Surgery, Faculty of Medicine, Justus-Liebig-University of Giessen, Gießen, Deutschland

**Keywords:** Rheumatoide Arthritis, Osteoporose, Rotatorenmanschette, Regenerationsfähigkeit, Krankheitsaktivität, Rheumatoid arthritis, Osteoporosis, Rotator cuff, Regenerative capacity, Disease activity

## Abstract

Das Schultergelenk ist in einem hohen Prozentsatz in die Erkrankung einer rheumatoiden Arthritis (RA) einbezogen. Gleichzeitig entwickeln Patienten mit RA frühzeitig eine Osteoporose, die das Frakturrisiko erheblich ansteigen lässt. In den letzten Jahren hat sich bei niedriger Krankheitsaktivität und guter medikamentöser Einstellung die Möglichkeit des gelenkerhaltenden Vorgehens mit winkelstabilen Implantaten eröffnet. Diese Methode ist allerdings von der Frakturmorphologie abhängig und führt, nicht ausreichend berücksichtigt, zu einer hohen Rate an Folgeoperationen. Bei der Rekonstruktion der Rotatorenmanschette (RM) zeigen neuere Daten ermutigende Ergebnisse der Regenerationsfähigkeit, was nicht zwangsläufig eine sofortige inverse Schulterprothese indiziert. Die Nachbehandlung muss der verzögerten ossären Heilung Rechnung tragen.

## Abstract (en)

The shoulder joint is involved in a high percentage of rheumatoid arthritis (RA) cases. At the same time, patients with RA develop osteoporosis at an early stage, which significantly increases the risk of fracture. In recent years, joint-preserving procedures with stable-angle implants have become available for individuals with low disease activity and good medication control. However, this approach is dependent on the fracture morphology and, if not adequately addressed, is associated with a high rate of revision surgery. In rotator cuff reconstruction, recent data show encouraging results regarding regenerative capacity, which does not necessarily indicate immediate reverse shoulder arthroplasty. Follow-up treatment must consider delayed osseous healing.

Die Einbeziehung des Schultergelenks (humeroglenoidal) in die entzündlichen Veränderung bei einer rheumatoiden Arthritis (RA) ist im Vergleich mit der Normalbevölkerung deutlich erhöht. Die 10-Jahres-Inzidenz beträgt im Verlauf der Erkrankung bis zu 83 % [7, 25].

Durch eine frühzeitig gestellte Diagnose und suffiziente medikamentöse Therapie scheint der Anteil der Schulterbeteiligung gegenüber der Vor-Biologika-Ära gesunken zu sein, doch sollte sie bei den regelmäßigen Kontrollen immer abgefragt werden.

Zwischen der Beeinträchtigung des Schultergelenks durch die Erkrankung und dem Erleben der sich ergebenden resultierenden funktionellen Ausfälle besteht eine zeitliche Diskrepanz. Die entzündlichen Veränderungen an Synovialis, Bursen und den Sehnen der Rotatorenmanschette führen zu einer progredienten Zerstörung der Gelenke und der Weichteilstrukturen [5]. Bei einer fortgeschrittenen Omarthritis bleibt meist nur der endoprothetische Gelenkersatz als verbleibende Therapiemöglichkeit. Durch die langjährige systemische Einnahme von nichtsteroidalen Antirheumatika (NSAR) werden von Rheumatikern Schmerzen, Schwellungen und Bewegungseinbußen der betroffenen Schulter lange toleriert und spät realisiert, auch weil Patienten frühzeitig unbewusst Schon- und Ausgleichsbewegungen ausführen [34]. Die Einnahme von Glukokortikoiden erhöht die Wahrscheinlichkeit einer Osteoporose und nachfolgenden osteoporotischen Fraktur um das 1,6fache, weshalb es in den letzten Jahren zu einer Anpassung der Therapieempfehlungen gekommen ist [1, 20].

Meist stehen für die Betroffenen Beeinträchtigungen anderer Gelenke – z. B. an Händen und Füßen – im Vordergrund. Neben den zu erwartenden arthritischen Destruktionen der Gelenkflächen führen die entzündlichen Schädigungen des Muskelmantels zu gravierenden Störungen in der Beweglichkeit [5, 25]. Bei über 70-jährigen Rheumatikern ist daher in über 80 % mit Schäden an der Rotatorenmanschette zu rechnen [6, 30].

Fallen bei der Untersuchung und der Funktionsprüfung Defizite auf, sind weitergehende bildgebende Verfahren zur Diagnosesicherung indiziert, um durch eine adäquate, zeitnahe, stadienadaptierte Therapie die Funktion der Schulter zu erhalten [15]. Als schnelles und preiswertes Diagnosetool bietet sich hierfür die Sonographie an. An dieser Stelle sei auf den Beitrag aus der Arbeitsgruppe von Frau Prof. Ohrndorf in diesem Heft verwiesen. Zeigen sich Veränderungen der Rotatorenmanschette oder randständige Usuren, ergänzt die Magnetresonanztomographie (MRT) die Diagnostik, um Aussagen einer möglichen fettigen Degeneration und Schädigung der Muskeltrophik der Rotatoren zu ermöglichen. Lim et al. konnten in ihrer Studie nachweisen, dass zwischen Rheumatikern und Nichtrheumatikern keine Unterschiede bezüglich operativer Versorgung, Heilung und postoperativem Outcome bestehen [16]. Besteht eine fettige Degeneration der rupturieren Rotatorenmanschette galt bislang diese als nicht regenerationsfähig sowie reparabel und indizierte die Versorgung mit einer inversen Schulterprothese [13]. Neuere Therapieansätze nach Bandscheibenvorfällen und der immobilitätsbedingten Atrophie der Muskulatur könnten auch bei Rekonstruktionen der Rotatoren Optionen eröffnen [32]. In den letzten Jahren hat die Entwicklung der Prothetik zu einer Verbesserung der Standzeiten geführt, von der auch Rheumatiker profitieren. Dennoch gilt die Aussage von van de Stande, dass Diagnose, Pathologie und Prothesenspezifika signifikante Prädiktoren für die Ergebnisse der Versorgung sind [35].

Neben den inflammatorischen Veränderungen der periartikulären Gewebe lässt sich bei Rheumatikern bereits in sehr frühen Stadien der Erkrankung eine Beeinträchtigung des Knochenstoffwechsels mit einer sich entwickelnden Osteoporose nachweisen. Die Knochenmasse und -festigkeit nehmen bei Rheumatikern gegenüber der Normalbevölkerung bereits viele Jahre früher deutlich ab. Die Einnahme von Glukokortikoiden verstärkt die Umbauprozesse der Osteoporose zusätzlich. Verorteten Larsen, Dale und Eek (LDE) den Beginn der Osteoporose ab Stadium 3, so zeigen Arthro-CTs (Computertomographien) und hochauflösende MRTs die frühzeitige periartikuläre Osteoporose bereits weitaus früher, als nativradiologisch erfassbar (LDE) [15].

Das Schultergelenk, insbesondere der proximale Humerus, ist bei Osteoporose anfällig für gelenknahe Frakturen (Indexfraktur) [17].

Bei Hinweisen auf eine Osteoporose sollte, so nicht vorhanden, die Knochendichte bestimmt und eine adäquate Therapie eingeleitet werden [31]. Dies hat Auswirkungen auf die Versorgung inklusive der möglichen Augmentation des Knochens durch Knochenersatzmaterialien [23].

Häufig war und ist bei proximalen Humerusfrakturen die primäre Therapie konservativ, da die obere Extremität als nicht lasttragend gilt und Defizite in Achse, Länge und Rotation eher verziehen werden. Erst in den letzten Jahren gewinnt eine frühe, stabile Versorgung des proximalen Humerus für die Gesamtmobilität des Patienten an Bedeutung [17]. Entsprechend steigt die Zahl der operativen Versorgungen an, was auch der Alterung der Bevölkerung geschuldet ist [27].

Besteht der Verdacht auf das Vorliegen einer proximalen Humerusfraktur muss diese mittels Bildgebung verifiziert, klassifiziert oder ausgeschlossen werden. Da Rheumatiker mit erniedrigter Knochendichte hierbei durch das Raster zu fallen drohen, weil sie scheinbar zu jung erscheinen und das Trauma nicht ausreichend erscheint, müssen die behandelnden Ärztinnen und Ärzte darauf sensibilisiert werden und Patienten nicht vorschnell abweisen [11, 14].

Zur Diagnosesicherung und bei der Frage nach der operativen Versorgung einer proximalen Humerusfraktur erfolgt neben den Röntgenbildern in mindestens 2 Ebenen inzwischen fast standardmäßig die computertomographische Untersuchung (CT). Neben der Erfassung komplexer Frakturformen mit Informationen zu Größe und Lage der einzelnen Fragmente liefert sie Aussagen zur Gelenksituation und eventuell vorhandener Begleitverletzungen von Glenoid oder Coracoid [4]. Die für die differenzialtherapeutischen Überlegungen hilfreiche Bildgebung der Weichteile (Sonographie, MRT) ist meist durch einen schmerzhaften Bewegungsverlust und dadurch bedingte Einschränkungen der Untersuchung eingeschränkt.

Neben der radiologischen Einteilung nach Larsen et al. unterscheidet Hirooka verschiedene schulterspezifische Destruktionsformen, die auch eine Aussage über die geeignete endoprothetische Versorgung zulassen [8, 15]. Durch den medizinischen Fortschritt in der Therapie der RA bedeutet eine proximale Humerusfraktur beim Rheumatiker mit einer suffizienten Basistherapie und niedrigen Werten im DAS28 (Disease Activity Score-28) nicht automatisch eine endoprothetische Versorgung des Schultergelenks. So können bei frühzeitiger Diagnosestellung und Einleiten einer suffizienten Basistherapie deutlich mehr Betroffene eine Remission erreichen, was auch Auswirkungen auf den Knochenstoffwechsel bzw. die Knochendichte hat. Die früher verbreitete primär konservative Therapie mit Ruhigstellung und Konsolidierung und sekundärer endoprothetischer Versorgung für Rheumatiker kann für den Patienten erhebliche Auswirkungen in Bezug auf die Selbstversorgung und Unabhängigkeit darstellen.

Die sich ergebende Frage lautet, ob bei der Diagnose einer Fraktur des proximalen Humerus bei RA bei nachgewiesener Osteoporose eine gelenkerhaltende Osteosynthese gerechtfertigt und vertretbar erscheint. Oder ist die endoprothetische Versorgung im Hinblick auf eine sichere Mobilisation des Patienten zu favorisieren? Bislang gibt es hierzu keine evidenzbasierte Therapieempfehlung. Ist eine Osteoporose im nativen Röntgenbild erkennbar (LDE Stadium 3), muss auch mit einer Beeinträchtigung der Gelenkflächen gerechnet werden, was dann die Empfehlung des endoprothetischen Gelenkersatzes favorisiert.

## Operative Versorgung

Das primäre Ziel einer operativen Versorgung ist die Wiederherstellung einer weitgehend physiologischen Schulterfunktion. Dies setzt eine anatomische Reposition und stabile Osteosynthese bzw. Endoprothese und die Möglichkeit der frühfunktionellen Nachbehandlung voraus. Neben der zumeist schlechten Knochenqualität beeinflussen bei Rheumatikern das Stadium der Omarthritis mit Synovialitis und der Zustand der Rotatorenmanschette bzw. bei Humeruskopffrakturen der Tuberkula und die Situation am Glenoid die Wahl der Versorgung. Für Rheumapatienten mit einer niedrigen Krankheitsaktivität oder in Remission ist eine gelenkerhaltende Versorgung ein Option und nicht per se ausgeschlossen. Erlaubt die Osteosynthese mit einer winkelstabilen Platte den Gelenkerhalt, so muss meistens auf eine additive Synovialektomie verzichtet werden, um nicht die fragilen Durchblutungsverhältnisse zusätzlich zu kompromittieren.

**Abb. 1:**
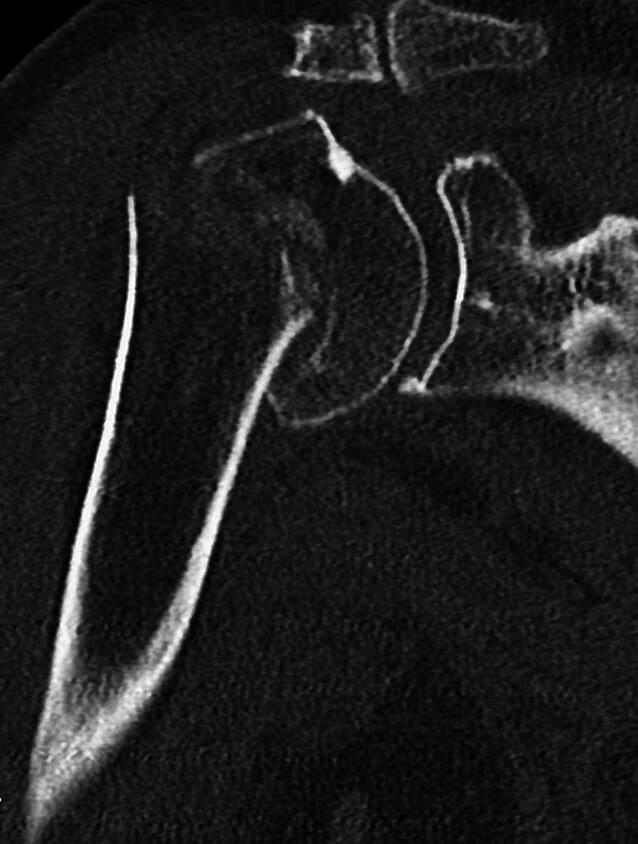
Computertomographie einer mehrfragmentären Humeruskopffraktur rechts bei Osteoporose, koronale Ebene. (© UKGM Gießen)

Die Fraktur des Humeruskopfes bei RA zeigt häufig neben der deutlich rarefizierten osteoporotischen Spongiosa subchondral nahe Frakturen mit fehlender ossärer Substanz, die eine sichere Verankerung der winkelstabilen Schrauben beeinträchtigt oder verhindert (Abb. 1).

**Abb. 2:**
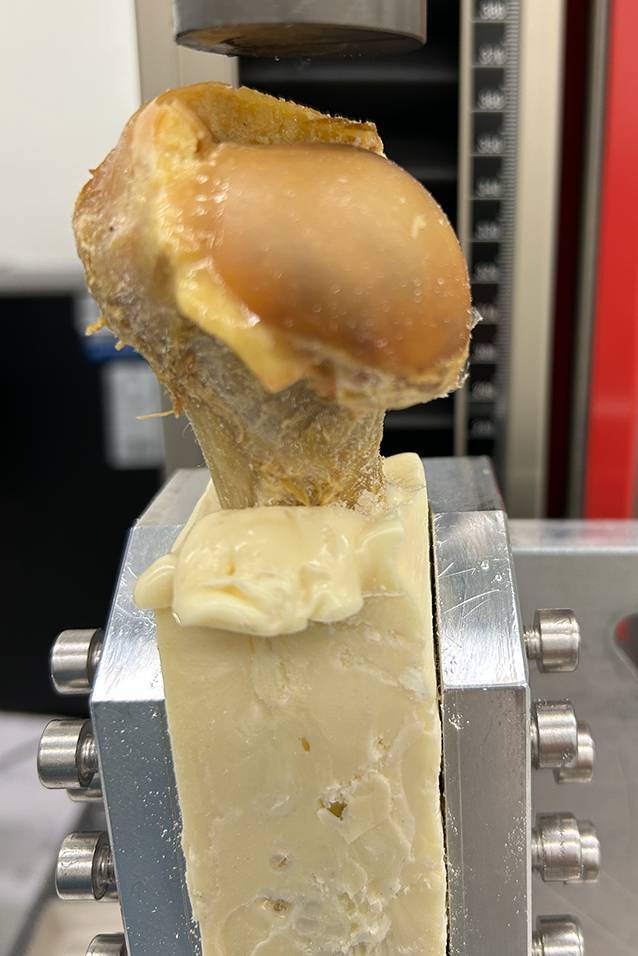
Biomechanische Testung auf Druck. Humeruskopf eines Körperspenders, Schaftlänge 11 cm (Zwick Roell, zwickiLine 5 kN zwicki; software: testXpert III; Ulm, Germany). (© C. Biehl)

**Abb. 3:**
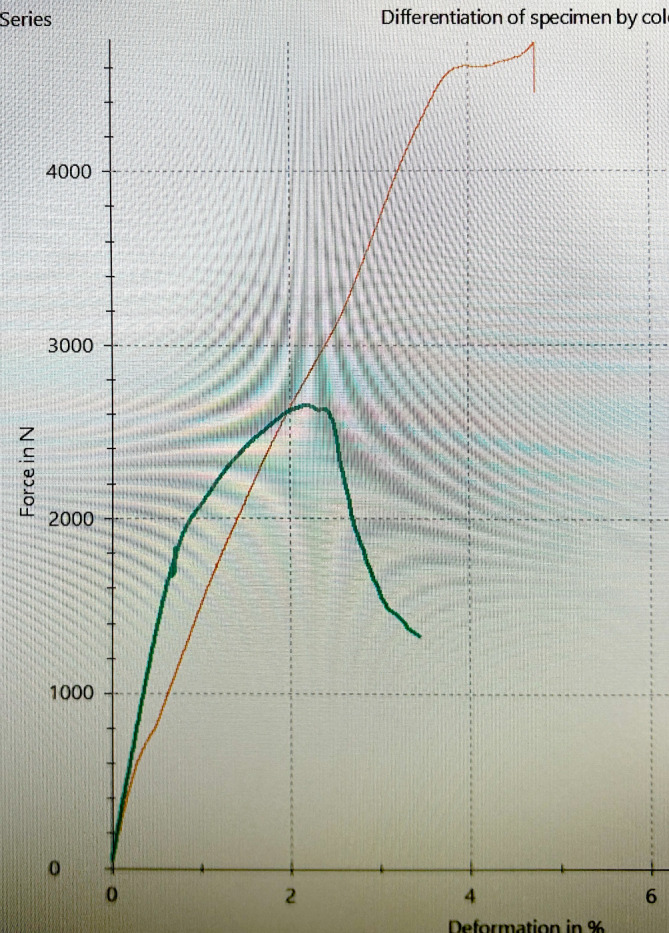
Exemplarische Druckkurven von humanem Knochen (*dunkelgrüne Linie*) und Sawbone® (*orangene Linie*). (© C. Biehl)

Doch auch bei ausreichendem Volumen des Kopfes ist der Anteil sekundärer Komplikationen erhöht. Nicht selten sintert der Humeruskopf sekundär in eine Valgusposition mit der Gefahr, dass die Schrauben in die Gelenkfläche ein- oder durchbrechen. Bei biomechanischen Tests an proximalen Humeri von Körperspendern zeigte sich bei osteoporotischen Humerusköpfen eine deutlich verminderte Resistenz gegenüber Druck im Vergleich zu Humeri von nichtosteoporotischem Knochen oder Sawbone® (Sawbone®, PACIFIC RESEARCH LABORATORIES, INC., Vashon, WA, USA) (Abb. 2). Im direkten Vergleich zeigte sich im Vergleich zu der einwirkenden Kraft, die zur Fraktur eines nichtosteoporotischen Humerus führt, bereits bei zwei Drittel der einwirkenden Kraft eine Fraktur. Hingegen lag die erforderliche Kraft beim Sawbone® deutlich jenseits von 4500 N (Abb. 3). Der Probenumfang war für statistisch relevante Aussagen zu klein; das Vorliegen einer RA war nicht bekannt.

Um das Risiko sekundärer Komplikationen zu reduzieren und eine stabile Verankerung auch bei osteoporotischem Knochen zu gewährleisten, haben die Hersteller von Osteosynthesesystemen reagiert und diese an die Erfordernisse adaptiert. Inwieweit die bei manchen Systemen vorhandenen aufsteigenden Schrauben am Collum chirurgicum ein sekundäres Abkippen des Kopfes in eine Valgusposition verhindern, wurde im Labor in einem weiteren experimentellen Setting mit proximalen Humeri im 3D-Druckverfahren, Osteosynthese und anschließender biomechanischer Testung als Vorstudie überprüft. Neben der Schraubenposition und der Möglichkeit der Augmentation des Schraubenlagers wurde auch die Rigidität der Implantate diskutiert. Aktuell werden verschiedene Knochenersatzmodelle erarbeitet, die in den biomechanischen Eigenschaften näher an den humanen Knochen herankommen als Sawbone®, um eine bessere Aussagefähigkeit zu verschiedenen Settings (Knochendichte, Osteosynthesen etc.) zu erreichen.

Die sich entwickelnden postarthritischen (oder sekundär arthrotischen) Veränderungen an den Gelenkflächen lassen sich meist nur zeitlich verzögern und führen langfristig zu Schmerzen und einem Funktionsdefizit der Schulter. Bei einem operativen gelenkerhaltenden Vorgehen erfolgte bis Anfang der 2000er-Jahre bei der offenen Synovialektomie des Schultergelenks zur Schmerzreduktion meist eine Denervation nach Baumann am proximalen Humerus [3]. Im Rahmen der Operationen hin zu primär arthroskopischen Verfahren kommt diese Technik nur noch sporadisch vor.

**Abb. 4:**
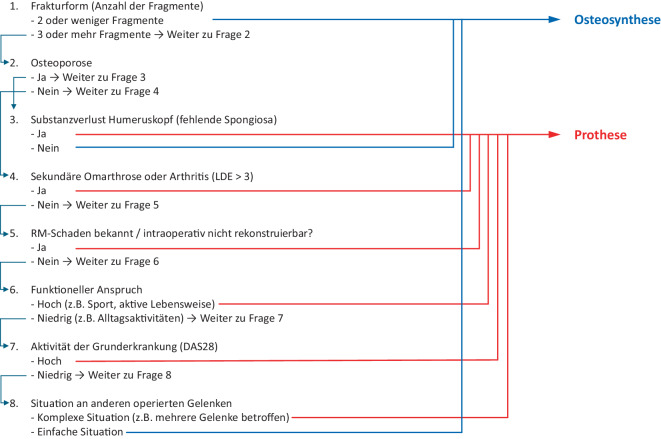
Entscheidungshilfe für die Versorgung von Frakturen des Humeruskopfes bei Patienten mit Osteoporose/rheumatoider Arthritis. *LDE* Larsen, Dale und Eek, *RM* Rotatorenmanschette, *DAS28* Disease Activity Score-28

Auch wenn sich die Situation der Rheumatiker seit Einführung und Etablierung der Biologikatherapie in der Fläche, in Verbindung mit einer früheren Diagnose und Therapieeinleitung verbessert hat, scheint die Osteosynthese mit winkelstabilen Implantaten am proximalen Humerus bei diesen Patienten weiterhin nicht die primäre Therapie der Wahl. Im Vorfeld können/sollten bereits verschiedene Parameter, die die Versorgung und das spätere Outcome beeinflussen, abgeklärt werden. So kann die Wahrscheinlichkeit eines intraoperativen Wechsels der Versorgung reduziert werden (Abb. 4).

Studien mit einer statistisch relevanten Zahl an Rheumatikern mit gelenkerhaltenden Versorgungen sind selten, in der eigenen Klinik wurden zwischen 2019 und 2022 87 Patienten mit einer Humeruskopffraktur osteosynthetisch mit winkelstabiler Platte versorgt. Lediglich 3 Patienten wiesen zum Zeitpunkt der Operation eine gesicherte RA als Grunderkrankung auf, sodass keine statistisch belastbaren Aussagen zum gelenkerhaltenden Vorgehen bei der kleinen Subgruppe möglich sind. Dagegen waren 29 Patienten älter als 75 Jahre und wiesen eine erniedrigte Knochendichte auf. Das Durchschnittsalter betrug knapp 64 Jahre (16 bis 90 Jahre). Insbesondere bei älteren Patienten mit gesicherter Osteoporose verbleiben häufig funktionelle Einschränkungen nach Osteosynthese. Die Ergebnisse nach Hemiprothese des Schultergelenks sind vergleichbar und beruhen hier entweder auf einem Substanzverlust des Glenoids oder auf einer prekären Weichteilsituation [28]. Karimi et al. haben eine Multicenterstudie aufgelegt, bei der die osteosynthetische Versorgung am Humerusschaft der konservativen Therapie gegenübergestellt wird [12].

Wie bei der endoprothetischen Versorgung gilt auch bei der Osteosynthese die Rekonstruktion der Höhe des Kopfes, des lateralen Offsets und der Retroversion. Meist liegen allerdings bei Rheumatikern mehrfragmentäre Frakturen mit Einbeziehung der Tuberkula und ein osteoporotisch ausgehöhltes Kalottenfragment vor, das gegen einen Erhalt des Gelenks und für den endoprothetischen Ersatz spricht.

Die endoprothetische Versorgung wird als Schaft-verankertes Implantat mit Rekonstruktion der Tuberkula und den oben beschriebenen Einstellungen vorgenommen. Aufgrund der rarefizierten Spongiosa und bei frakturierten Tuberkula empfiehlt sich die zementierte Verankerung des Prothesenschafts im Humerus. Da sich in den letzten Jahren eine deutlich verbessere Schmerzsituation durch die gleichzeitige Versorgung des Glenoids nachweisen lässt und die Verankerung der Implantate verbessert wurde, sollte eine Totalendoprothese implantiert werden. Rüther und Niemeier weisen in ihrem Kapitel zur Schulter darauf hin, dass bei Rheumapatienten das Glenoid entgegen dem des Arthrotikers ventral und kranial destruiert, was bei der Ausrichtung der Prothese berücksichtigt werden muss [26]. Mit Implantation der Prothese kann gleichzeitig die offene Synovialektomie erfolgen. Die viele Jahre eingesetzten Kappenprothesen sind nahezu gänzlich vom Markt verschwunden, Duokopf- oder Großkopfprothesen behalten als Rückzugsmöglichkeit bei Versagen einer (inversen) Schulterprothese eine Daseinsberechtigung [34].

## Nachbehandlung

Bei 3‑ oder 4‑Fragment-Frakturen mit Beteiligung der Tuberkula müssen diese nach erfolgreicher Rekonstruktion und eventueller Spongiosaplastik die Möglichkeit der ossären Integration erhalten. Eine forcierte frühfunktionelle physiotherapeutische Beübung steht dem häufig entgegen. Die Nachbehandlungsempfehlungen der Deutschen Gesellschaft für Orthopädie und Unfallchirurgie (DGOU) geben hier Empfehlungen für die postoperative Therapie, sind aber noch keine allgemein anerkannten Richtlinien (Tab. 1; [29]). In der Klinik werden daher Patienten mit einer mehrfragmentären Fraktur postoperativ anfangs wie bei einer konservativen proximalen Humerusfraktur nachbehandelt. Bei sich abzeichnender ossärer Heilung erfolgt die Mobilisation der Schulter analog zur Rekonstruktion der Rotatorenmanschette, wobei auf das Roll-Dreh-Gleiten des Humeruskopfes gegenüber dem Glenoid mit Eintauchen des Kopfes in Richtung kaudaler Recessus geachtet werden muss. Das Konzept der propriozeptiven neuromuskulären Fazilitation (PNF) dient der Optimierung und Ökonomisierung der Bewegung und ermöglicht daher auch bei prekären Gelenksituationen die Verbesserung der Schultersituation. Entscheidend ist die gute Zusammenarbeit zwischen Patient, Operateur und Physiotherapie.

**Tab. 1:** Postoperative Therapie einer proximalen Humerusfraktur bei Osteoporose, angelehnt an die Nachbehandlungsempfehlungen der Deutschen Gesellschaft für Orthopädie und Unfallchirurgie (DGOU) und Burkhart et al. [4, 29]

Woche	Therapie
1	Thorax-Abduktions-Kissen (6 Wochen, zur Entlastung der Tuberkula), Beübung von Hand und Ellenbogen
2–3	Pendelübungen, Mobilisation Skapula
4–6	Aktiv-assistive Physiotherapie bis 90° Abduktion und Elevation
Ab 7. Woche	Nativ-Röntgen, ggf. Computertomographie und Freigabe bei gesicherter Konsolidierung

## Diskussion

Frakturen des proximalen Humerus bei Rheumatikern weisen gegenüber Arthrosepatienten einige Besonderheiten auf. Neben der Arthritis in Gelenk und Bursen sind frühzeitig die Sehnen der Rotatorenmanschette in die Erkrankung einbezogen [18, 25]. Zusätzlich weisen die meisten Rheumatiker eine Osteopenie und Osteoporose auf, die gegenüber Arthrotikern um Jahrzehnte vorausgehen kann [9]. Diese führt bei Frakturen des proximalen Humerus meist zu einer kalottennahen Bruchzone, die die Verankerung winkelstabiler Schrauben erschwert. Die Schrauben müssen nahe an die subchondrale Schicht platziert werden, um den Kopf zu fassen. Bei einem sekundären Verlust der Gelenkflächenposition kommt es zu einem erhöhten Risiko einer Penetration der Schraubenenden durch die Gelenkfläche. Das Versagen der Osteosynthese und die Revisionsrate bei osteoporotischen Frakturen betragen bis zu 30 % [21]. Daher sehen die meisten orthopädischen Rheumatologen eine osteosynthetische Versorgung kritisch. Andererseits haben die Fortschritte in der medikamentösen Behandlung inflammatorischer Erkrankungen bei Ansprechen der Therapie zu einer dem Arthrotiker vergleichbaren Knochensituation geführt [22]. Die flächendeckende Etablierung von Biologika und die Anpassung der Diagnose- und Therapierichtlinien seit 2000 hat bei Neuerkrankungen zu einer Verbesserung auch der ossären Strukturen geführt [19]. Allerdings gilt dies nur bei Patienten mit Neudiagnose und einer Verträglichkeit der Medikation. Folglich sind bei solchen Patienten gelenkersetzende Endoprothesen allein aus der Tastsache des Vorliegens einer RA nicht obligat. Auch wenn die primär prothetische Versorgung einer proximalen Humerusfraktur einfacher erscheint, muss der veränderten Anatomie bzw. pathologischen Prozessen der rheumatischen Schulter Rechnung getragen werden [18]. Neben einer gegenüber Arthrotikern vermehrten Retroversion müssen frakturierte Tuberkula sicher refixiert werden. In der postoperativen Nachbehandlung muss die verzögerte ossäre Heilung berücksichtigt werden, sodass sowohl bei der Osteosynthese als auch bei der endoprothetischen Versorgung zunächst die Prämisse Stabilität vor Mobilität gelten sollte. Iking et al. fanden in ihrer Metaanalyse zwischen den konservativen und operativen Verfahren keine signifikanten, klinisch bedeutsamen Unterschiede zwischen der Lebensqualität oder den Schmerzen bei älteren Patienten [10]. Südkamp et al. konnten in ihrer prospektiven Multicenterstudie für die Osteosynthese zeigen, dass ein gutes funktionelles Ergebnis auf die richtige Operationstechnik zurückzuführen ist, Rheumatiker wurden hier allerdings nicht gesondert erfasst und ausgewertet [33].

Der Erfolg der Operation ist primär von der Qualität und Rekonstruierbarkeit der Weichteilstrukturen, insbesondere der Rotatorenmanschette und der postoperativen Nachbehandlung abhängig [16, 24]. Neuere Studien weisen bei RA auf zufriedenstellende Ergebnisse und eine erforderliche Revisionsrate von rund 10 % nach 4 bis 5 Jahren hin [2]. Neben der Weichteilsituation wird die Wahl der Versorgung vom knöchernen Destruktionsgrad an Humerus und Glenoid und dem erwarteten Spontanverlauf beeinflusst. Die Verankerung der Prothesen, insbesondere des glenoidalen Teils, hat in den letzten Jahren eine stetige Verbesserung erfahren, was selbst bei schwierigen Verhältnissen eine Versorgung ermöglicht. Paras et al. fanden in ihrer Metaanalyse aus 200 Arbeiten vergleichbare Ergebnisse in Scores, hingegen war das funktionelle Outcome inverser Schulterprothesen bei Frakturen schlechter als bei elektiven Operationen [24].

## Limitationen

Die eingangs erwähnte Studie soll Aussagen zur biomechanischen Situation von proximalen Humerusfrakturen in Beziehung zum klinischen Outcome ermöglichen und in einer Subgruppenanalyse auch Aussagen bei osteoporotischem und inflammatorischem Knochenstatus bei Frakturen aufzeigen. Sie ist in mehreren Punkten eingeschränkt. Erstens berichten die Autoren von einer sehr kleinen und zufällig ausgewählten Stichprobe. Zweitens wurde nur eine einmalige Belastung mit dem Drucksystem durchgeführt, die jedoch nicht vollständig der Alltagsbelastung entspricht. Die Ergebnisse der biomechanischen Tests der verschiedenen Hersteller zum Vergleich wären sicherlich hilfreich. Drittens steht eine klinische Überprüfung der in der Klinik erfolgten Versorgungen mithilfe der Standard-Scores und dem „patient related outcome measurement“ (PROM) wegen Auflagen der Statistiker noch aus.

## Fazit für die Praxis

- Rheumatiker haben eine erhöhte Gefahr, eine proximale Humerusfraktur zu erleiden. Dies ist in der Erkrankung selbst und der verbreiteten Glukokortikoidtherapie begründet.
- Die Versorgung richtet sich auch nach der Aktivität der Erkrankung und der Destruktion der Rotatorenmanschette.
- Bei Patienten mit manifester Osteoporose und mehrfragmentärer Fraktur ist bei einem Erhaltungsversuch mit Osteosynthese mit einer hohen Rate an Revisionen zu rechnen.
- Osteosynthesen mit winkelstabilen Implantaten sind für Patienten mit niedriger Krankheitsaktivität und guten Knorpelverhältnissen eine Option.
- Bei Osteosynthesen ist die Synovialektomie des Gelenks erschwert.
- Die endoprothetische Versorgung ist bei gleichzeitigen Glenoiddefekten eher anzustreben.

## Data Availability

Die in dieser Studie erhobenen Datensätze können auf begründete Anfrage beim Korrespondenzautor angefordert werden.

## References

[1] Abtahi S, Driessen JHM, Burden AM et al (2021) Concomitant use of oral glucocorticoids and proton pump inhibitors and risk of osteoporotic fractures among patients with rheumatoid arthritis: a population-based cohort study. Ann Rheum Dis 80:423–431 33310727 10.1136/annrheumdis-2020-218758

[2] Austin DC, Wilbur RR, Rogers TH et al (2023) Rotator cuff repair in patients with inflammatory arthritis: satisfactory midterm outcomes. JSES Int 7:30–34 36820413 10.1016/j.jseint.2022.08.019 PMC9937845

[3] Biehl C (2011) Die operative Therapie der rheumatischen Schulter. Akt Rheumatol 36:118–122

[4] Burkhart KJ, Dietz SO, Bastian L et al (2013) The treatment of proximal humeral fracture in adults. Dtsch Ärztebl Int 110:591–597 24078839 10.3238/arztebl.2013.0591 PMC3785018

[5] Deshmukh AV, Koris M, Zurakowski D et al (2005) Total shoulder arthroplasty: long-term survivorship, functional outcome, and quality of life. J Shoulder Elbow Surg 14:471–479 16194737 10.1016/j.jse.2005.02.009

[6] Gohlke F (2009) Defektarthropathie – sekundäre Omarthrose. Orthopädie und Unfallchirurgie up2date 4, S 121–136

[7] Hämäläinen M (1995) Epidemiology of upper limb joint affections in rheumatoid arthritis. In: Baumgartner H, Dvorak J, Grob D, Munzinger U, Simmen BR (Hrsg) Rheumatoid arthritis. Thieme. Thieme, Stuttgart New York, S 158–161

[8] Hirooka A, Wakitani S, Yoneda M et al (1996) Shoulder destruction in rheumatoid arthritis Classification and prognostic signs in 83 patients followed 5–23 years. Acta Orthop Scand 67:258–263 8686464 10.3109/17453679608994684

[9] Hooyman JR, Melton LJ 3rd, Nelson AM et al (1984) Fractures after rheumatoid arthritis. A population-based study. Arthritis Rheum 27:1353–1361 6508860 10.1002/art.1780271205

[10] Iking J, Fischhuber K, Stolberg-Stolberg J et al (2023) Quality of Life and Pain after Proximal Humeral Fractures in the Elderly: A Systematic Review. Medicina 59:10.3390/medicina59101728 PMC10608543 37893445

[11] Jin S, Hsieh E, Peng L et al (2018) Incidence of fractures among patients with rheumatoid arthritis: a systematic review and meta-analysis. Osteoporos Int 29:1263–1275 29546507 10.1007/s00198-018-4473-1

[12] Karimi D, Brorson S, Midtgaard KS et al (2022) Surgical versus non-surgical treatment of humeral SHAFT fractures compared by a patient-reported outcome: the Scandinavian Humeral diAphyseal Fracture Trial (SHAFT)-a study protocol for a pragmatic randomized controlled trial. Trials 23:453 35655280 10.1186/s13063-022-06317-6 PMC9161482

[13] Kasten P, Loew M (2007) How to treat massive rotator cuff tears. Orthopade 36:855–861 17704906 10.1007/s00132-007-1137-9

[14] Kovalenko PS, Dydykina IS, Postnikova PS et al (2023) Predictors of repeated and first-time low-energy fractures in patients with rheumatoid arthritis. Meditsinskiy Sovet = Med Counc: 136–144

[15] Larsen A, Dale K, Eek M (1977) Radiographic evaluation of rheumatoid arthritis and related conditions by standard reference films. Acta Radiol Diagn 18:481–491 10.1177/028418517701800415 920239

[16] Lim SJ, Sun JH, Kekatpure AL et al (2017) Rotator cuff surgery in patients with rheumatoid arthritis: clinical outcome comparable to age, sex and tear size matched non-rheumatoid patients. Ann R Coll Surg Engl 99:579–583 28853601 10.1308/rcsann.2017.0107 PMC5697045

[17] Neuerburg C, Schneller J, Kammerlander C (2020) Frakturen im Alter. Orthopädie und Unfallchirurgie up2date 15, S 219–233

[18] Niemeier A, Ruther W (2011) Shoulder arthroplasty for primary synovial diseases. Z Rheumatol 70(380):382–387 10.1007/s00393-011-0769-7 21698477

[19] O’dell JR (2002) Treating rheumatoid arthritis early: a window of opportunity? Arthritis Rheum 46:283–285 11840429 10.1002/art.10092

[20] Ochi K, Furuya T, Ishibashi M et al (2016) Risk factors associated with the occurrence of proximal humerus fractures in patients with rheumatoid arthritis: a custom strategy for preventing proximal humerus fractures. Rheumatol Int 36:213–219 26420406 10.1007/s00296-015-3371-5

[21] Olerud P, Ahrengart L, Ponzer S et al (2011) Internal fixation versus nonoperative treatment of displaced 3‑part proximal humeral fractures in elderly patients: a randomized controlled trial. J Shoulder Elbow Surg 20:747–755 21435907 10.1016/j.jse.2010.12.018

[22] Ozen G, Pedro S, Wolfe F et al (2019) Medications associated with fracture risk in patients with rheumatoid arthritis. Ann Rheum Dis 78:1041–1047 31092411 10.1136/annrheumdis-2019-215328

[23] Pankratz C, Dehner C, Gebhard F et al (2024) Augmentation techniques for the treatment of osteoporosis-associated fractures of the extremities. Unfallchirurgie 127:253–262 38351179 10.1007/s00113-024-01414-4

[24] Paras T, Raines B, Kohut K et al (2022) Clinical outcomes of reverse total shoulder arthroplasty for elective indications versus acute 3‑ and 4‑part proximal humeral fractures: a systematic review and meta-analysis. J Shoulder Elbow Surg 31:e14–e21 34454040 10.1016/j.jse.2021.07.014

[25] Petersson CJ (1986) Painful Shoulders in Patients with Rheumatoid Arthritis: Prevalence, Clinical and Radiological Features. Scand J Rheumatol 15:275–279 3798043 10.3109/03009748609092592

[26] Rehart S, Sell S, Arbogast M et al (2015) Expertise Orthopädische Rheumatologie. Thieme

[27] Rupp M, Walter N, Pfeifer C et al (2021) The Incidence of Fractures Among the Adult Population of Germany—an Analysis From 2009 through 2019. Dtsch Ärztebl Int 118:665–669 34140088 10.3238/arztebl.m2021.0238 PMC8727861

[28] Schill S, Thabe H, Grifka J (2002) Differential therapy for the rheumatoid shoulder. Orthopade 31:1132–1144 12486539 10.1007/s00132-002-0401-2

[29] Schmidt J, Riedel T, Grundler S et al (2024) DGOU Nachbehandlungsempfehlungen 2024

[30] Schwyzer H, Gschwend N, Simmen B (1994) Zur Häufigkeit der Rotatorenmanschettenruptur bei der cP-Schulter. Akt Rheumatol 19(05):134–135

[31] Singh A, Adams AL, Burchette R et al (2015) The effect of osteoporosis management on proximal humeral fracture. J Shoulder Elbow Surg 24:191–198 25240809 10.1016/j.jse.2014.07.005

[32] Spang C, Golonka W, Domokos B et al (2024) Reduzierung der pathologischen Verfettung in der autochthonen Rückenmuskulatur durch Isoliertes Widerstandstraining – eine prospektive MRT Studie an chronischen Rückenpatienten. VSOU, Baden-Baden, S 76–154

[33] Südkamp N, Bayer J, Hepp P et al (2009) Open reduction and internal fixation of proximal humeral fractures with use of the locking proximal humerus plate. Results of a prospective, multicenter, observational study. J Bone Joint Surgery Am 91:1320–1328 10.2106/JBJS.H.00006 19487508

[34] Thabe H, Brackertz D (1997) Praktische Rheumaorthopädie. Chapman and Hall

[35] Van De Sande MA, Brand R, Rozing PM (2006) Indications, complications, and results of shoulder arthroplasty. Scand J Rheumatol 35:426–434 17343249 10.1080/03009740600759720

